# Isolation of *Streptococcus agalactiae* and an aquatic birnavirus from doctor fish *Garra rufa* L

**DOI:** 10.1186/2046-0481-66-16

**Published:** 2013-09-13

**Authors:** Neil M Ruane, Evelyn M Collins, Michelle Geary, David Swords, Cathy Hickey, Fiona Geoghegan

**Affiliations:** 1 Fish Health Unit, Marine Institute, Rinville, Oranmore, Co. Galway, Ireland

**Keywords:** Aquabirnavirus, *Streptococcus agalactiae*, *Garra rufa*

## Abstract

**Background:**

The doctor fish, *Garra rufa*, has become increasingly popular as a treatment for skin disorders and for pedicures in recent years. Despite this there is very little information available regarding the welfare of these fish and the range of potential pathogens they may carry. In this study, a group of fish suffering from post-transport mortalities were examined and the isolated pathogens identified.

**Findings:**

Group B *Streptococcus agalactiae* was isolated from kidney swabs of the fish and found to be resistant to a number of antibiotics. In addition to this, a fish virus belonging to the aquabirnavirus group, serogroup C was isolated for the first time in Ireland. However, no clinical signs of disease typical of bacterial or viral infections were observed in any fish examined.

**Conclusions:**

As no clinical signs of disease attributable to either of the pathogens identified were found it was concluded that the mortalities were most likely due to transport related stress exacerbated by the presence of the pathogens. Further work is required to assess the suitability of current transport strategies and to examine the potential risk associated with the transport of live ornamental fish.

## Background

*Garra rufa* (Heckel, 1843), commonly known as doctor fish, is a non-migratory, freshwater species belonging to the carp family (*Cyprinidae*). The native range of *G. rufa* is from the Persian Gulf to the eastern Mediterranean, encompassing all the major river basins in that region [1]. Doctor fish are benthic feeders, known to adhere by suction to rocks while feeding on plant material using a modified lower lip termed ‘mental adhesive disc’ [2]. It is this feeding behaviour which led to the use of these fish as treatments for skin disorders such as psoriasis for many years, most notably in Turkey where the practice was first described in 1989 [3]. Although the exact mechanisms are not known, there is some scientific evidence which suggests that the use of these fish may be beneficial for people with skin problems [4,5]. In recent years, this has been capitalised on by the health spa industry with a proliferation of ‘foot spas’ offering treatments using doctor fish which in turn has placed a high demand for their supply. Transport of ornamental fish is characterised by high loading densities, high levels of metabolic waste in the transport water and post-transport mortality due to the stressful nature of the procedure [6]. In addition to this, the movement of live fish is often one of the main risk factors associated with the spread of any disease [7]. To date, there are very few reports describing pathogens or disease in *G. rufa* and such information is needed for the effective analysis of any risk associated with the transport of large numbers of these fish. This report describes the isolation of the bacteria *Streptococcus agalactiae* and an aquatic birnavirus from *G. rufa* submitted to the laboratory by a health spa when high levels of mortality were noted after delivery.

## Methods

In August 2011, a health spa reported a number of fish dead on arrival after receiving a batch of *G. rufa* and that mortalities continued to occur over the following days after placing the fish in a holding tank. Thirty of these fish were brought to the Fish Health Unit laboratory, Marine Institute, Galway and samples were taken for routine bacteriological, virological and histological examination. Initial examination showed that the fish were lethargic and no external parasites were observed following skin and gill scrapes. Kidney swabs from five separate fish were plated onto tryptone soya agar (TSA) and Colombia blood agar (CBA) plates and incubated at 22, 30 and 37°C. Growth of bacterial colonies was identified on the basis of standard phenotypic testing criteria (Gram stain, motility, oxidase activity). Sequencing of the partial 16S ribosomal RNA gene was also used to confirm the identity of the bacteria [8] and serotyping was carried out using a commercial kit (Oxoid). Sensitivity of the bacteria to a range of antibiotics was also performed. For virus isolation, fifteen fish (three pools of five) were homogenised and inoculated onto bluegill *Lepomis macrochirus* fry (BF-2) and epithelioma papulosum cyprinid (EPC) cell lines and incubated at 20°C. When cytopathic effect was noted on the cell lines, the virus was identified using commercial ELISA and IFAT kits (TestLine and BioX Diagnostics) and by nested RT-PCR [9]. For histology, ten whole fish were fixed in neutral-buffered formalin and processed according to standard methods. Sections were cut at 5 μm and stained with haematoxylin-eosin.

**Figure 1:**
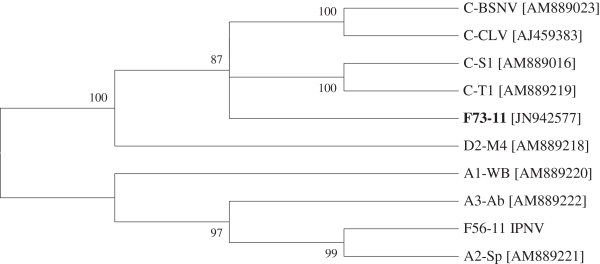
**A condensed phylogenetic tree showing relationships between the *Garra rufa *birnavirus F73-11 and other aquatic birnaviruses from serogroups A, C and D (indicated as a prefix), based on nucleotide sequence comparisons of a 226 bp segment of the VP1 protein.** The tree was constructed using the neighbour-joining method and 1000 bootstrap replicates were performed for each analysis to assess the likelihood of the tree construction. Only values > 70 are indicated. [GenBank accession numbers are shown in brackets].

PCR products were visualised after electrophoresis on a 1.5% (w/v) agarose gel in TAE buffer (40 mM Tris, 20 mM acetic acid, 2 mM ethylenediaminetetraacetic acid) stained with ethidium bromide, with the Quantity One 1-D Analysis System software on a UV Transluminator (Bio-Rad). The PCR products were purified and sequenced commercially (Sequiserve). The virus isolate identified in this study was given the reference IRL-F73-11 and compared with nine other birnavirus isolates (Figure 1). These included a known infectious pancreatic necrosis virus isolate (IRL-F56-11) isolated from a marine Atlantic salmon farm, representatives of IPNV serogroups A1, A2 and A3 and five isolates representing serogroups C and D [8]. Multiple sequence alignments were performed by Clustal W analysis and all phylogenetic analyses were conducted using MEGA 5 [10].

## Results

The characteristics of the bacteria isolated are listed in Table 1. The consensus sequence for the partial 16S ribosomal RNA gene was subjected to a BLAST search (http://blast.ncbi.nlm.nih.gov/Blast.cgi) and was found to have 100% homology with *Streptococcus agalactiae*. Serotyping further confirmed that it was a Group B *Streptococcus*. Optimal growth was observed at 30°C, with slow growth at 22°C and no growth at 37°C. Of the six antibiotics used for the sensitivity testing, no zones of inhibition were recorded for flumequine, oxolinic acid, sarafloxacin and trimethoprim/sulphamethoxazole suggesting that the bacteria were resistant to these antibiotics. Some sensitivity to amoxicillin and oxytetracycline was noted with zones of 5 and 10 mm diameters recorded respectively.

**Table 1:** **Characteristics of the bacteria isolated from *Garra rufa *and identified as *Streptococcus agalactiae***

**Characteristic**	
Gram stain	Positive bacilli
Morphology	Small white circular colonies
Motility	Negative
O/F	Inert
Oxidase	Negative
Haemolysis	β
Growth at 22°C	Yes (slow)
Growth at 30°C	Yes
Growth at 37°C	No
Amoxycillin (10 μg)	Sensitive
Flumequine (4 μg)	Resistant
Oxolinic acid (2 μg)	Resistant
Oxytetracycline (30 μg)	Sensitive
Sarafloxacin (5 μg)	Resistant
Trimethoprim/Sulphamethoxazole (25 μg)	Resistant

Cytopathic effects were noted on the BF2 cell lines following the first passage after seven days, however the EPC cell line was unaffected. Tests using commercial ELISA and IFAT kits for viral haemorrhagic septicaemia (VHS), infectious pancreatic necrosis (IPN), infectious haematopoietic necrosis (IHN) and spring viraemia of carp (SVC) viruses yielded negative results. Phylogenetic analysis of the partial sequence of the viral protein 1 (VP1) gene showed that the virus was closely related to serogroup C and D aquabirnaviruses (Figure 1). The analysis also showed that the *G. rufa* birnavirus was assigned to the clade including blotched snakehead virus (BSNV), the type species for *Blosnavirus*. All selected isolates of serogroup A, which have IPNV as the type species, grouped together in one clade, although the assay was able to differentiate each of the serotypes (A1–A3) and grouped the isolate, IRL-F56-11, together with serotype A2.

After histological examination of the internal organs, no pathology associated with bacterial or viral infection was observed.

## Discussion

This study reports the first isolation of *Streptococcus agalactiae* and an aquatic birnavirus from the doctor fish, *G. rufa* in Ireland. As the fish examined in this study did not show clinical signs of disease, the cause of the mortality was most likely due to transport stress exacerbated by the presence of both pathogens. However it should be noted that acute mortalities can often occur following viral/bacterial infections with little or no clinical signs of disease. The increased popularity of health spas using these fish for pedicures has resulted in a dramatic rise in the demand for their supply. A 2012 report from the UK [11] stated that between 15,000–20,000 *G. rufa* were imported each week through Heathrow Airport, the main border inspection post for the import of live fish into the UK. Despite the large numbers of fish coming into the EU, there is very little published information available on the requirements of these fish, either in terms of fish welfare (transport, husbandry etc.), or in terms of the risk they pose to native fish stocks and to customers who use these fish at health spas. Mortality of *G*. *rufa* in fish hatcheries has been associated with the aquatic pathogens *Aeromonas sobria*[12] and *Citrobacter freundii*[13]. This study reported the isolation of *S. agalactiae* from moribund fish without clinical signs of disease, although it is known that this bacteria may be pathogenic for fish species including *G. rufa*[11,14]. These bacteria, also known as Lancefield group B streptococcus, have a broad host range and can cause meningoencephalitis in fish, mastitis in cattle and meningitis in human neonates. Although there is a high genetic diversity between strains of *S. agalactiae*, experimental transmission studies have shown that isolates from human, bovine and piscine origins can cause clinical disease in fish [15,16]. A range of other bacteria with the potential to cause zoonotic infections have been isolated from doctor fish [11] including vibrio and mycobacteria species. As these bacteria do not have optimal growth at 37°C, infections are mostly confined to the superficial, cooler body tissues of the extremities. Reports of infections are rare however and have mostly been confined to fish handlers and aquarium hobbyists [17], however at least one infection due to a pedicure treatment has been reported [18]. The UK Health Protection Agency has published guidelines for the management of health risks associated with fish pedicures and has classified the risk of zoonotic infection as low [19]. The *G*. *rufa* isolate was also found to be resistant to four out of six antibiotics tested and the susceptibility to the remaining two antibiotics is questionable due to the small size of the inhibition zones and the general lack of data in which to calculate epidemiological cut-off values for determining sensitivity. Antimicrobial resistance of bacteria isolated from tropical fish is well known due to the widespread use of antibiotics in the transport water [11,20].

The birnavirus family are double-stranded, non-enveloped RNA viruses consisting of four genera, two of which contain aquatic viruses, *Aquabirnavirus* (type species: infectious pancreatic necrosis virus IPNV) and *Blosnavirus* (type species: blotched snakehead virus BSNV). Aquabirnaviruses have been isolated from aquatic animals throughout the world and infectious pancreatic necrosis is a significant disease of farmed salmonids in Ireland [21]. Although this is the first isolation in Ireland of an aquatic birnavirus, other than IPNV, mortalities due to birnavirus infections in tropical fish have been reported previously [10,22]. Sequence analysis indicated that the isolate was similar to other viruses, such as BSNV, isolated from tropical fish [9] and initially assigned to a separate serogroup (C) within the genus *Aquabirnavirus*. Studies on BSNV [23] showed that it was distinct from other aquabirnaviruses and was assigned to its own genus, *Blosnavirus*[24]. Although further characterisation of the virus isolate from this study is required, the results would suggest that the birnavirus isolated from *G*. *rufa* is more related to blosnavirus than aquabirnavirus.

## Conclusion

The transport of live aquatic animals is a significant risk factor in the spread of disease [7]. The ornamental fish industry is an expanding sector with large volumes of fish traded annually [25]. It is a concern for a trading block such as the European Union, that imported animals and animals traded within the Community could potentially pose a human health risk. Council Directive 2006/88/EC, which currently regulates the movements of live fish into and within Europe, focuses on the control of diseases of fish health significance rather than of public health significance. In fact, it has been suggested that ornamental fish represent a special case in live animal trade due to the large number of fish species originating from numerous sources, together with a lack of knowledge of the potential pathogens which they may carry [25]. This is supported by the detection of the pathogens described in this study, in sub-clinically infected fish, highlighting the risk of transmission to other aquarium fish and a need for the establishment of improved quarantine and pathogen screening procedures for fish such as *G. rufa* which are to be used in the health sector.

## Competing interests

The authors declare that they have no competing interests.

## Authors’ contributions

NR carried out the molecular analysis, sequence alignment and drafted the manuscript. EC performed the histopathology analysis and helped to draft the manuscript. MG performed the bacteriology analysis. DS performed the virological analysis. CH performed the histological analysis. FG helped to draft the manuscript and gave final approval of the version to be published. All authors read and approved the final manuscript.
